# Clearance of the SARS-CoV-2 Virus in an Immunocompromised Patient Mediated by Convalescent Plasma without B-Cell Recovery

**DOI:** 10.3390/ijms22168902

**Published:** 2021-08-18

**Authors:** Maamoun Basheer, Elias Saad, Orly Laskar, Ofir Schuster, Hagai Rechnitzer, Simona Zisman-Rozen, David Azoulay, Nimer Assy

**Affiliations:** 1 Internal Medicine Department, Galilee Medical Center, Nahariya 2210001, Israel; EliasS@gmc.gov.il; 2 Azrieli Bar-Ilan Faculty of Medicine, Safad 2210001, Israel; DavidA@gmc.gov.il; 3 Department of Infectious Diseases, Israel Institute for Biological Research, Ness Ziona 7404905, Israel; orlyl@iibr.gov.il (O.L.); schustero@gmc.gov.il (O.S.); 4 The Clinical Microbiology Laboratory, Galilee Medical Center, Nahariya 2210001, Israel; HagaiR@gmc.gov.il (H.R.); Zisman-Rozen@gmc.gov.il (S.Z.-R.); 5 Hematology Unit and Clinical Laboratory, Galilee Medical Center, Nahariya 2210001, Israel

**Keywords:** immunocompromised, SARS-COV-2, pneumonia, T lymphocytes

## Abstract

Coronavirus disease (COVID-19) is a contagious disease caused by the severe acute respiratory syndrome coronavirus 2 (SARS-CoV-2). This case report presents a patient who had difficulty eradicating the corona virus due to being treated with Rituximab, which depletes B lymphocyte cells and therefore disables the production of neutralizing antibodies. The combined use of external anti-viral agents like convalescent plasma, IVIG and Remdesivir successfully helped the patient’s immune system to eradicate the virus without B-cell population recovery. In vitro studies showed that convalescent plasma is the main agent that helped in eradicating the virus.

## 1. Introduction

Coronavirus disease (COVID-19) is contagious and is caused by the severe acute respiratory syndrome coronavirus 2 (SARS-CoV-2) [1]. Rituximab, an anti-CD20 monoclonal antibody, is one of the main treatments for B-cell malignancies and auto-immune diseases [2]. It acts by depleting normal cells (as well as pathogenic B cells) while sparing other plasma cells and hematopoietic stem cells [2,3]. This medication prolongs B-cell depletion, which impairs the adaptive immune response and the ability to produce neutralizing antibodies. Patients treated with Rituximab are at a higher risk for prolong severe forms of COVID-19 [4,5,6,7].

This case report presents a patient treated with Rituximab, and discusses the specific interventions conducted to eradicate the virus.

## 2. Material and Methods

### 2.1. Convalescent Plasma

Convalescent plasma donations were tested for the presence of anti-SARS-CoV-2 IgG using an ELISA assay with the spike protein as an antigen. The titer of anti-SARS -CoV-2 IgG was more than 1:1000. The protocol of the Israeli ministry of health is two units of convalescent plasma for COVID-19 patients [8].

### 2.2. Flow Cytometry Analysis of Leucocyte Differentials and Lymphocytic Subsets

For measuring lymphocyte sub-set proportions, 50 µL of whole blood was incubated with an antibody mixture containing: Pacific-Blue conjugated anti-human CD7 (Beckman Coulter Inc., Carlsbad, CA, USA), PE-Cy7 conjugated anti-human CD45 (Beckman Coulter Inc., Carlsbad, CA, USA), PE-Cy5, conjugated anti-human CD56 (Beckman Coulter Inc., Carlsbad, CA, USA), ECD conjugated anti-human CD3 (Beckman Coulter Inc., Carlsbad, CA, USA), PE conjugated anti-human CD8 (Beckman Coulter Inc., Carlsbad, CA, USA) and FITC conjugated anti-human CD4 and CD19 (Beckman Coulter Inc., Carlsbad, CA, USA). The samples were incubated for 10 min at room temperature, and underwent red blood cell lysis via VersaLyse solution (Beckman Coulter Inc., Carlsbad, CA, USA)for an additional 10 min. The total lymphocytes count of the patients were determined by the system XN-1000 hematology analyzer (Sysmex Corporation, Kobe, Japan) and recorded. The results were read by a Beckman Coulter Navios flow cytometer. The total leucocytes were recorded, and the percentages of the lymphocyte subsets from the total gated lymphocytes were also recorded.

### 2.3. COVID-19 Infection Status as Detected by the Nasopharyngeal RT-PCR Test and the Cytopathic Effect

Following nasal and throat sampling, viral swabs were inserted into refrigerated transfer buffer containing tubes (Copan). Tubes containing the swabs were vortexed for 1 min. Two ml of the buffer were transferred to a new 15mL tube and centrifuged (5000× *g*, 5 min, 4 °C), and the supernatant was transferred through a 0.22 µm filter. Vero E6 (ATCC CRL-1586TM), were cultured in DMEM supplemented with 10% fetal bovine serum (FBS), MEM non-essential amino acids, 2mM L-Glutamine, 100 Units/mL Penicillin, 0.1 mg/mL streptomycin, 12.5 Units/mL Nystatin (Biological Industries, Beit Haemek, Israel). Each supernatant sample was added in duplicates to cells monolayers in 12-well plates (Costar; 0.2 mL/well) for 1 h, followed by the addition of 2 mL MEM containing 2% FBS, MEM non-essential amino acids, 2 mM L-Glutamine, 100 Units/mL Penicillin, 0.1 mg/mL streptomycin, 12.5 Units/mL Nystatin and 0.15% sodium bicarbonate (Biological Industries, Beit Haemek, Israel). Plates were further incubated at 37 °C, 5% CO_2_ for 5 days. SARS-CoV-2 (GISAID accession EPI_ISL_406862), kindly provided by Bundeswehr Institute of Microbiology, Munich, Germany, was used as positive control at a concentration of 60 pfu/mL. Cytopathic effect (CPE) was microscopically determined [9]. To confirm that the cytopathic effect was due to SARS-Cov-2, a RT-real time PCR for the virus was performed on cell supernatant. The assay was conducted on days 41, 54 and 95 after the first positive corona test.

RT-PCR and PCR were used for SARS-CoV-2 detection. Viral RNA was extracted from nasopharyngeal samples using the QIAamp Viral RNA Mini Kit (Qiagen) according to the manufacturer’s instructions. RT-PCR and PCR were performed subsequently with a one-step real time RT-PCR kit containing primers and probes targeting the ORF1b and a positive internal reference gene. Reaction system and amplification conditions were performed according to the manufacturer’s specifications (Wuhan BGI Biotechnology Co. Ltd., Wuhan, China). The result was considered valid only when the cycle threshold (Ct) value of the reference gene was ≤32. The result was considered positive for 2019-nCoV when the Ct value of the ORF1b target gene was ≤36 (borderline result was determined when the Ct value of the ORF1b target gene was >36 but ≤40 and negative when ORF1b Ct was above 40 or not detected).

### 2.4. In Vitro Studies of the Efficiency of Different Agents to Eradicate the SARS-CoV-2 Virus

SARS-CoV-2 viruses (60 PFU/well) were treated with different materials (Remdesivir 100 µg/mL, Ivermectin 3 µg/mL, IVIG 20 mg/mL or convalescent plasma 50 µL/well). The blood of the patient was drained to an EDTA tube. The wells were then incubated with 50 µL buffy coat, the fraction of an anti-coagulated blood sample that contained most of the white blood cells of the patient. Each mixture was transferred to Vero E6 cells, followed by 5 days of incubation. The cytopathogenic effect of the intact virus was measured in all study groups.

### 2.5. Ethics

This work was done according to the instructions of the local committee of Helsinki for human Studies. The patient signed a form concern confirming the publication of this work.

## 3. Results

### 3.1. Clinical and Labortory Monitoing of the Pateint

**Figure 1:**
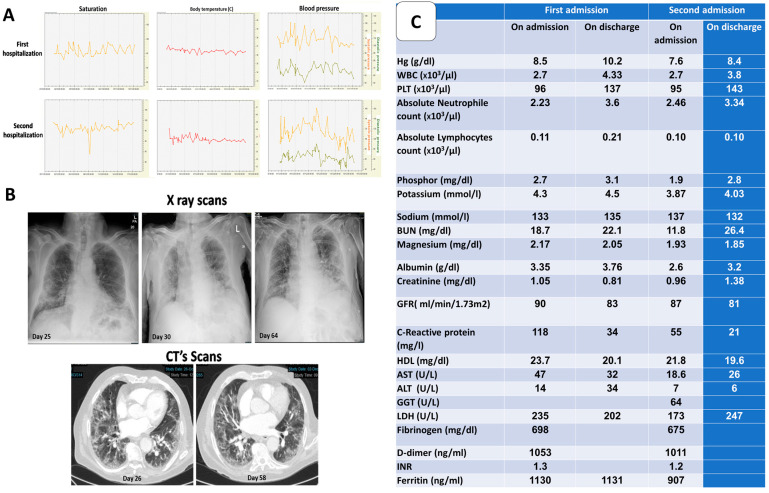
(**A**) O_2_ saturation, body temperature, systolic and diastolic blood pressure. The upper and lower panels relate to the first and the second hospitalization, respectively. (**B**) Upper panel: Posterior anterior chest radiograph showing bilateral patchy lung consolidation in a peripheral distribution pattern on days 25, 30 and 64 after the first positive corona test. Lower panel: Unenhanced, thin-section CT performed on day 26 after the first positive corona test showing bilateral ground–glass attenuations with subsequent deterioration in the second hospitalization on day 58. (**C**) Complete blood count and bio-chemical results of the patient during the first and the second hospitalizations upon admittance and discharge.

The patient was infected with the SARS-CoV-2 virus on day 1. On day 25, he came to the emergency room with general weakness, cough and dyspnea. The RT-PCR from a nasopharyngeal sample was positive for corona virus. While hospitalized in a designated COVID-19 ward, the patient was treated with two units of convalescent plasma, systemic steroids, anticoagulants and vitamin D, a known COVID-19 treatment protocol, with a good response. Throughout the hospitalization, the patient was hemodynamically stable with normal saturation levels on room air (Figure 1A). Radiographic imaging showed about 50% of lung injury Figure 1B). He was discharged on day 41 with no need for supplementary oxygen.

On day 61, the patient returned to the hospital with a fever. Laboratory tests showed signs of inflammation. Repeated SARS-CoV-2 RT-PCR were positive, causing concern for a continuous persistent COVID-19 infection. The patient was therefore, readmitted to the COVID-19 ward. To achieve virus eradication, the patient underwent a second regiment of Remdesivir (10-day course), four units of convalescent plasma, 120 g of intravenous immunoglobulin and one dose of ivermectin (15 mg). The patient improved and was discharged within 10 days.

The complete blood count of the patient showed thrombocytopenia and severe lymphopenia (Figure 1C). Biochemical analysis showed stable plasma electrolyte concentrations. Inflammatory parameters (C-reactive protein, ferritin, D-dimer and fibrinogen) were increased upon admission. A decrease in these parameters was noted once treatment was presented (Figure 1C).

### 3.2. Analysis of Leucocytes Differential and Lymphocyte Subsets Proportion by Flow Cytometry

**Figure 2:**
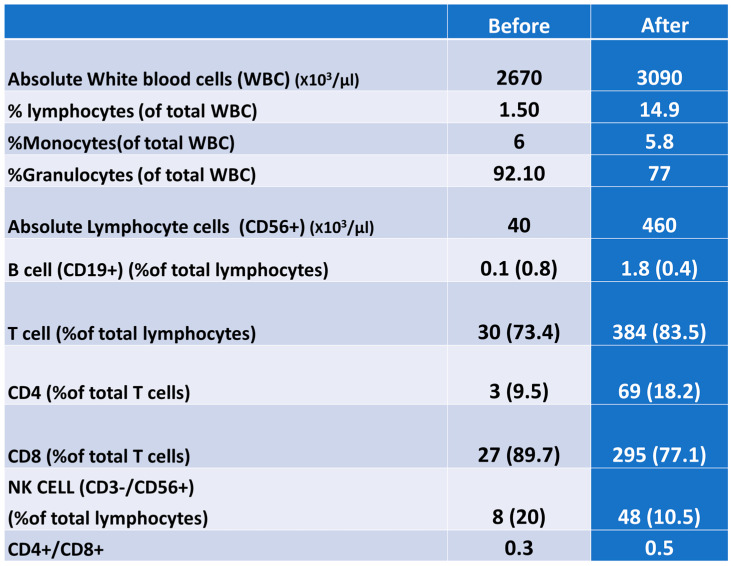
Analysis of leucocytes differential and lymphocyte subsets proportion by flow cytometry.

Analysis of the leucocyte differential and lymphocyte subset proportion via flow cytometry showed a depletion of B-cells (<1%) upon admission with no recovery after the intervention. However, T-cells were increased after intervention from 73% to 83.5%. The CD4, CD8 and NK cell (CD3-/CD56+) populations also increased after the treatment. The CD4+/CD8+ ratio was low before the intervention, with no further changes (Figure 2).

### 3.3. COVID-19 Infection and Immunity Status as Detected by a Nasopharynx COVID-19 PCR Test and Viral Culture

COVID-19 infection and immunity status were tested. RT-PCRs were performed on nasal and throat samples on days 1, 25, 41, 54, 60, 75, 95 and 136, after the first positive corona test was done. To confirm that the virus was alive and intact, the viral cytotoxic effect was tested on a VERO cell culture.

**Figure 3:**
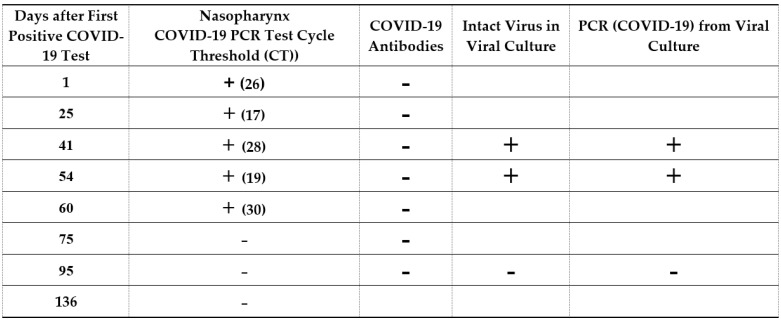
COVID-19 infection and immunity status as detected by nasopharynx COVID-19 PCR tests and viral culture. A PCR test of nasal and throat samples showed positive test on days 1, 25, 41, 54 and 60 with a low cycle threshold (CT). Negative results were visible on days 75, 95 and 136. Cell cultures showed intact viruses on days 41 and 54. Negative cell culture for the corona virus was verified on day 95 after the completed course of the treatment.

The results showed that the patient was positive for the Corona virus (days 1, 25, 41, 54 and 60) until intervention as shown also by the cytotoxic culture assay in days 41 and 54. Negative SARS-CoV-2 RT PCR results were initially achieved after the intervention (days 75, 95 and 136). A cytotoxic culture assay confirmed the eradication of the virus after intervention (day 95). No SARS-CoV-2 antibodies were found (Figure 3). Viral genetic sequencing showed that it was probably a wild strain and less suitable for variant sequencing.

### 3.4. In Vitro Studies: Different Agents’ Effects on SARS-CoV2 Cytotoxic Effect Using Vero E6 Cells

**Figure 4:**
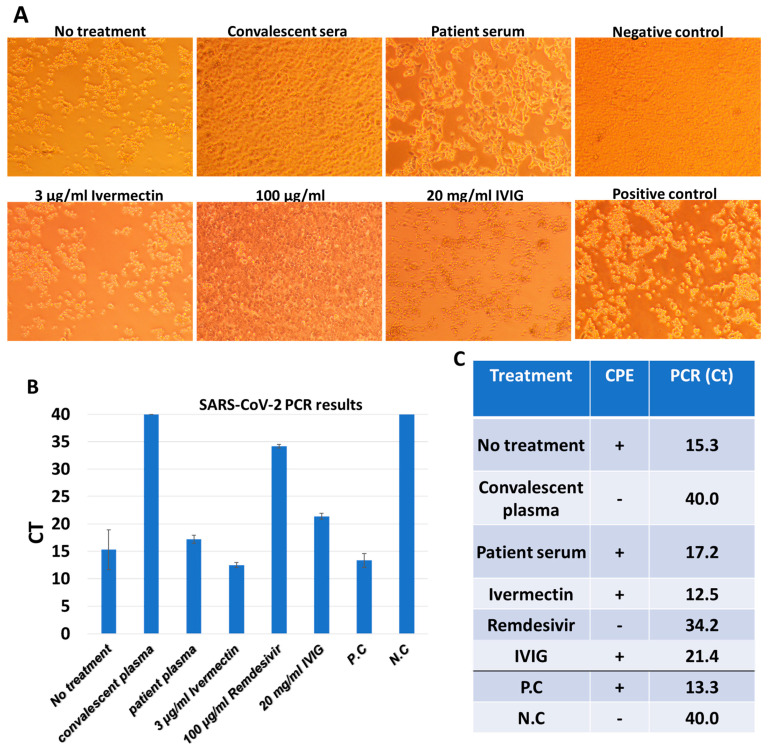
In vitro studies confirming that convalescent plasma and Remdesivir successfully eradicated the virus. (**A**): SARS-CoV-2 viruses (60 PFU/well) were treated with different agents in the presence of white blood cells from the immunodeficient patient. Each mixture was transferred to Vero E6 cells, followed by five days incubation to the formation of CPE. Representative images for each treatment are presented. (**B**): PCR results of SARS-CoV-2 identification from each treatment are presented (averages of three triplicates with their SD). P.C: positive control. N.C.: negative control. (**C**): Summary of CPE and PCR results for each treatment. +/− presence/absence of CPE.

In vitro studies confirmed that convalescent plasma and Remdesivir successfully eradicated the virus. Convalescent sera or Remdesivir were shown to confer protection against the cytopathic effect, while CPE was observed in all other treatments (Figure 4). The PCR results showed low or absent viral loads in convalescent plasma or Remdesivir samples. These data support the CPE observations, and demonstrates that CPE formation was due to SARS-CoV-2 presence (Figure 4).

All substances (Remdesivir, Ivermectin, IVIG and convalescent plasma) without patient blood cells were added to Vero E6 cells with or without SARS-CoV-2, and their effect on the cells was tested. It was found that in the absence of the virus, no CPE was observed using any of the substances. In the presence of SARS-CoV-2 and without patient blood cells, CPE observed in the Ivermectin and IVIG samples, whereas in the Remdesivir and convalescent plasma samples, minimal CPE was observed. The RT-PCR results for these samples showed that there was a significant eradication of the virus; however, at slightly lower CT levels (33 and 35, respectively) compared to these samples in the presence of white blood cells from the patient. This suggests that the patient immune system with convalescent plasma synergically eradicate the virus.

## 4. Discussion

This case report presents a patient treated with Rituximab, which depletes B lymphocyte cells and therefore disables the production of neutralizing antibodies, creating difficulties in eradicating the corona virus.

On day one, the infected patient was asymptomatic. He presented to the ER on day 25 with general weakness. His first hospitalization period extended to 16 days. The patient was treated with two units of convalescent plasma, systemic steroids, anti-coagulants and vitamin D, a known COVID-19 treatment protocol (as recommended by the Israeli ministry of health) with good response. He was discharged on day 41 with no need for supplementary oxygen. The RT-PCR assay from a nasopharyngeal sample was still positive, while anti–SARS specific- coV-2 IgG were negative.

Due to the fact that the patient was treated with Rituximab, he was immunocompromised, and due to the positive cell culture test, the patient was still contagious and was indicated for quarantine. From day 41 through day 61, the patient was quarantined alone at home. During this period, the patient felt good, with no need for supplementary oxygen. Another RT-PCR test taken on day 54, with positive results, with no SARS-CoV-2 antibodies. A cell culture test also showed that the patient still had live intact virus particles within the cells.

At day 61, the patient returned to the hospital with a fever. Repeated RT-PCR assays were positive, causing concern for a continuous persistent COVID-19 infection. During this hospitalization stay, the patient received a second course of Remdesivir (10-day course), four units of convalescent plasma, 120 g of intravenous immunoglobulin, as recommended by some studies [10,11,12] and one dose of Ivermectin (15 mg) [13]. He improved under this treatment. On discharge day (day 77), an RT-PCR assay from a nasopharyngeal swab was negative and no Coronavirus antibodies were found. Furthermore, a cell culture test was repeated laterally on day 95, with no presence of live intact viruses. It should be noted that analysis of whole blood samples taken both on admission and discharge, showed a limited B lymphocyte population (about 1%).

The patient was positive for COVID-19 for 75 days, confirmed by a special cell culture test that showed evidence of intact live virulent viruses with cytotoxic abilities. No anti-SARS-CoV-2 IgG antibodies were identified. A specific intervention, which included convalescent plasma and another extended course of Remdesivir, eventually eradicated the virus. Remdesivir and convalescent plasma are anti-viral agents, but each works through different mechanisms. Remdesivir is known to inhibit the replication of the virus. It works as a nucleoside analog and inhibits the RNA-dependent RNA polymerase. On the other hand, convalescent plasma contains SARS-CoV-2 antibodies, which work synergically with the patient immune system in order to eradicate the virus.

Rituximab treated patients need passive immunotherapy to eradicate viruses. The supply of these antibodies by convalescent plasma is critical for the good function of the immune system mediating viral clearance. The immune system, with B cell depletion, could eradicate the SARS-CoV-2 virus. These results were also previously presented by Thomas Hueso et al. [10]. This case study suggests that T cells and other cells in the innate system work synergistically with exogenous antibodies in eradicating the SARS-CoV-2 virus.

In conclusion, this case report presents a patient treated with Rituximab, which depletes B lymphocyte population and, therefore, disables the production of neutralizing antibodies. The combined use of external anti-viral agents such as convalescent plasma and Remdesivir, helped the patient’s immune system in eradicating the virus, with no need for B-cell population recovery.

## Data Availability

All data from patient file.
